# Effectiveness of a Proactive Mail-Based Alcohol Internet Intervention for University Students: Dismantling the Assessment and Feedback Components in a Randomized Controlled Trial

**DOI:** 10.2196/jmir.2062

**Published:** 2012-10-31

**Authors:** Preben Bendtsen, Jim McCambridge, Marcus Bendtsen, Nadine Karlsson, Per Nilsen

**Affiliations:** ^1^Department of Medical and Health SciencesFaculty of Health SciencesLinköping UniversityLinköpingSweden; ^2^Faculty of Public Health & PolicyLondon School of Hygiene and Tropical MedicineLondonUnited Kingdom; ^3^Department of Computer and Information ScienceInstitute of TechnologyLinköping UniversityLinköpingSweden

**Keywords:** Alcohol drinking, Web-based intervention, proactive intervention, university students

## Abstract

**Background:**

University students in Sweden routinely receive proactive mail-based alcohol Internet interventions sent from student health services. This intervention provides personalized normative feedback on alcohol consumption with suggestions on how to decrease drinking. Earlier feasibility trials by our group and others have examined effectiveness in simple parallel-groups designs.

**Objective:**

To evaluate the effectiveness of electronic screening and brief intervention, using a randomized controlled trial design that takes account of baseline assessment reactivity (and other possible effects of the research process) due to the similarity between the intervention and assessment content. The design of the study allowed for exploration of the magnitude of the assessment effects per se.

**Methods:**

This trial used a dismantling design and randomly assigned 5227 students to 3 groups: (1) routine practice assessment and feedback, (2) assessment-only without feedback, and (3) neither assessment nor feedback. At baseline all participants were blinded to study participation, with no contact being made with group 3. We approached students 2 months later to participate in a cross-sectional alcohol survey. All interventions were fully automated and did not have any human involvement. All data used in the analysis were based on self-assessment using questionnaires. The participants were unaware that they were participating in a trial and thus were also blinded to which group they were randomly assigned.

**Results:**

Overall, 44.69% (n = 2336) of those targeted for study completed follow-up. Attrition was similar in groups 1 (697/1742, 40.01%) and 2 (737/1742, 42.31% retained) and lower in group 3 (902/1743, 51.75% retained). Intention-to-treat analyses among all participants regardless of their baseline drinking status revealed no differences between groups in all alcohol parameters at the 2-month follow-up. Per-protocol analyses of groups 1 and 2 among those who accepted the email intervention (36.2% of the students who were offered the intervention in group 1 and 37.3% of the students in group2 ) and who were risky drinkers at baseline (60.7% follow-up rate in group 1 and 63.5% in group 2) suggested possible small beneficial effects on weekly consumption attributable to feedback.

**Conclusions:**

This approach to outcome evaluation is highly conservative, and small benefits may follow the actual uptake of feedback intervention in students who are risky drinkers, the precise target group.

**Trial Registration:**

International Standard Randomized Controlled Trial Number (ISRCTN): 24735383; http://www.controlled-trials.com/ISRCTN24735383 (Archived by WebCite at http://www.webcitation.org/6Awq7gjXG)

## Introduction

University entrance is associated with increased levels of heavy drinking. Alcohol is often used to promote social integration despite the potential negative consequences of excessive drinking among students. A survey of more than 18,000 students aged 17 to 30 years in 21 countries found that substantial numbers of them used alcohol, ranging from 29% of men and 6% of women in South Africa to 95% of men and 93% of women in Ireland [1]. Young adult students in Sweden also show evidence of high alcohol consumption. A survey conducted with 4575 undergraduate university students on four campuses in Sweden found that 96% of students had consumed alcohol in the preceding 12 months [2]. A recent study surveyed 1585 first-year students in Linköping University and found that heavy episodic (binge) drinking was reported by 51% of the women and 71% of the men [3].

Several studies have assessed the effectiveness of various forms of Internet-based screening, combined with brief interventions, to change drinking behaviors, as shown for example by Boon et al [4] and Saitz et al [5]. A recent Cochrane review by Moreira and colleagues [6] compared the effectiveness of various forms of social norms interventions in university and college settings involving electronic screening and brief intervention (e-SBI), mailed feedback on pen-and-paper screening, individual face-to-face feedback, and group face-to-face feedback. Evidence indicates that e-SBI methods influence a wider range of factors related to alcohol habits, including peak blood alcohol concentration, drinking frequency, and drinking quantity, than other delivery methods and are less expensive to use [6].

The use of e-SBI has to a great extent emerged as an efficient approach to reaching large numbers of adolescents as a result of high levels of Internet use among young people. Advantages of the use of Internet-delivered interventions compared with face-to-face consultations include greater reach and implementation, higher consistency of intervention content, and closer matching of intervention to patient characteristics and recommended guidelines [7,8]. The psychometric properties of existing screening instruments when administered online have been found to be reliable [9].

Many earlier studies of e-SBI among university students have focused on the effectiveness of a normative feedback on students’ drinking behaviors [10-15]. A meta-analysis conducted by Carey et al [16] found that students receiving personalized normative feedback demonstrated significant reductions in harmful alcohol-related behaviors. Overall, personalized normative interventions appear to be effective in reducing alcohol use and related problems among university and college students when the student’s own drinking is compared with that of other students, of the same sex and age, at the same university or college.

More research is needed on many aspects of e-SBI in student populations. The majority of the research has been conducted in the United States. Many previous studies have required respondents to participate in e-SBIs taking place in controlled settings, rather than allowing students to access e-SBIs using their own computers [17,18]. Only a few published studies have described projects that made more comprehensive use of electronic media, by recruiting large numbers of participants via email, and having participants complete e-SBIs at their own convenience, using their own or others’ computers. A substantial number of e-SBI projects, including our own studies [3,19], have been more feasibility studies and were not performed as full-scale randomized controlled trials. One exception is several randomized controlled trials performed in Australia by Kypri et al, including a large-scale study of 13,000 university students [20].

Another area where more knowledge is needed concerns whether the assessment or screening of alcohol consumption per se reduces drinking [21-26]. Several reviews of brief alcohol interventions have noted unexpected levels of reductions in drinking among control groups [27,28]. Indeed it is not uncommon to find a 20% alcohol consumption reduction in control groups who do not receive an evaluated intervention. Such reductions have usually been explained by regression to the mean, the effects of natural variation in people’s drinking patterns, and by a possible inadvertent intervention effect of the assessment and research procedures [21]. If students can be influenced to reduce their drinking at hazardous or harmful levels with simple screening, large-scale implementation of screening surveys among university students might have a considerable public health impact, with no need for a more elaborate normative feedback intervention.

Alcohol screening involves answering a series of questions and is thus a form of brief assessment. A recently published systematic review of randomized evaluations of assessment effects in brief intervention found somewhat equivocal evidence of assessment reactivity when studies with adults, some of which identified no brief intervention effects, were included [26]. When attention was restricted to university students, however, stronger evidence was obtained, confirming small assessment effects as a result of answering questions in both interviews and self-completion questionnaires, including online questionnaires. These findings are relevant to the possibility of developing brief assessment-based interventions and also suggest that previous studies may have been biased by contamination of control groups. University students may possibly be more receptive to assessment effects than other populations.

This study addressed several of the identified gaps in previous research. The overall aim of this study was to evaluate the effectiveness of e-SBI, using an randomized controlled trial design that takes account of baseline assessment reactivity (and other possible effects of the research process) due to the similarity between the intervention and assessment content. The design of the study allowed exploration of the magnitude of the assessment effects per se. More specifically, the study tested 4 main hypotheses as follows:

1. Alcohol Use Disorders Identification Test (AUDIT) scores in the group that was assessed at baseline and received feedback (group 1) will be lower than in the group that was not contacted at baseline (group 3), providing a test of the effects of universal e-SBI provision in an unselected population of university students.

2. AUDIT scores in the baseline assessment-only group (group 2) will be lower than in the no-contact baseline group (group 3), providing a test of the effects of assessment-only in an unselected population of university students.

3. AUDIT scores among risky drinkers in group 1 will be lower than in group 2, providing a test of the effects of adding feedback to assessment-only among risky drinkers who participated at baseline.

4. AUDIT scores in group 1 as a whole will be lower than in group 2 as a whole, providing a test of the effects of adding feedback to assessment-only in an unselected population of university students

## Methods

### Study Setting and Population

The study was performed at Linköping University in the mid to southern part of Sweden among all 5227 freshmen entering the university. Every student at the university has a personal official university email address that is obligatory for the student to use, with all official mail delivered through this address. The e-SBI was performed by the individual students on personal computers at a location of their preference, usually at home.

### Design of the Study

**Figure 1:**
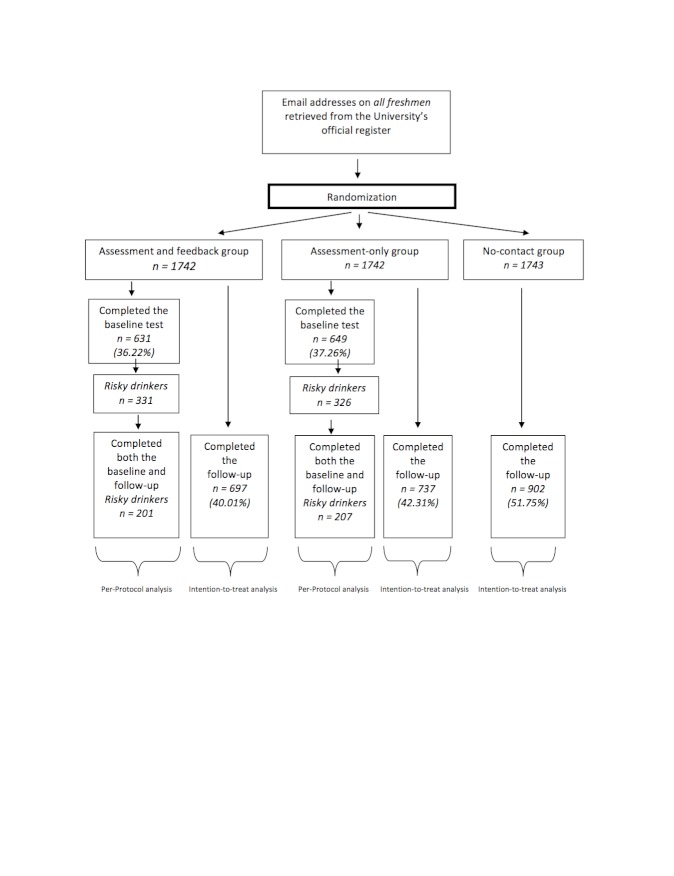
CONSORT flowchart for the study.

The study was performed as a three-arm parallel-groups trial in which routine provision of e-SBI (group 1) was compared with the same without feedback (ie, assessment-only; group 2) and no-contact control (group 3) study conditions. Groups 1 and 2 completed identical assessments, the sole difference between them being that group 1 was provided with normative feedback as usual, whereas participants in group 2 were simply thanked for their participation and given a link to a commonly used website concerning alcohol information with no normative feedback. Group 3 was contacted only after 2 months, at which time both groups 1 and 2 also completed outcome data collection (Figure 1).

The study was designed to protect the control group as much as possible from the possible effects of research participation [29]. Thus, participants in the control group were not aware that they were part of a trial (nor were groups 1 and 2) and were contacted only at the time of follow-up.

### Randomization and Other Study Procedures

We collected email addresses from the official university register in three separate data files. All participants had a 1 in 3 probability of allocation to any particular study condition. Randomization was computerized and did not use any strata or blocks. Each participant was given a random number between 0.0 and 1.0 with 5 decimals. For instance, participant A might be given number 0.12345 and participant B might get 0.54321. The list of participants was then sorted in descending order by this number, and the list was cut into 3 equal parts (or as equal as possible depending on the number of participants).

At baseline the students received an email from the student health care service with a short greeting welcoming them to the university followed by an invitation to perform the Internet alcohol intervention. All three groups were sent an email 2 months later by the Swedish principal investigator (PB). This mail made no reference to the previous email from the student health care services and comprised an invitation to participate in an online alcohol research survey, with inclusion in a lottery draw for 100 cinema tickets as a small incentive to increase participation. The alcohol study outcomes were derived from the 10-item AUDIT questionnaire [30] in this survey, with two reminders sent 1 week apart, also containing a link to the questionnaire.

### Blinding

Groups 1 and 2 were unaware that they were participating in a research study when they responded to the initial emails. Both groups were led to expect that these emails were provided as routine practice by the student health care center to help students think about their drinking. All three groups were unaware they were participating in an intervention study and that they had been randomly assigned to a study group. At follow-up, no explanation of the true nature of the study was given to students. Instead they were invited to participate in a seemingly unrelated student alcohol survey. As all study procedures were automated, the research team had no direct contact with study participants. The use of blinding and deception in this trial raised ethical issues. All students were subsequently offered the opportunity to provide feedback on their alcohol consumption at the time of the follow-up. Ethical approval for the study was given by the regional ethics committee in Linköping, Sweden (reference number: 2010/291-31).

### Description of the Computerized Alcohol Intervention

The study was based on an email-based Internet alcohol intervention (e-SBI) that has been developed by the Lifestyle Intervention Research group at Linköping University [3,19]. The computerized intervention is a fully automated single-session intervention and has been used for some years as part of routine practice in the great majority of Swedish universities. Consequently the system is stable, and no bug fixes or content changes were necessary during the trial period, and we observed no downtime of the system. The participants were given an opportunity to comment on the intervention by sending an email to the company that operates the intervention. During the trial period, fewer than 10 participants commented on the content of the intervention, giving mostly positive comments.

After students received the welcoming email from the student health care service, they were sent an invitation to perform the Internet alcohol intervention by clicking on a hyperlink to the test. Two reminders were sent 1 week apart to those who had not answered and thereafter the link was closed. The link could be used only once in order to ensure that each student performed the test only once. The test included questions about average consumption day by day in a typical week during the last 3 month, frequency of binge drinking, highest blood alcohol concentration in the last 3 months (calculated by the computer based on the student’s input), negative consequences related to alcohol, views on how much other students and peers drink, and questions about motivation to change. A demo version of the computerized test can be viewed at demo.livsstilstest.nu.

Group 1 students then got immediate feedback consisting of 3 statements summarizing their weekly consumption, their frequency of heavy episodic drinking, and their highest blood alcohol concentration during the last 3 months, comparing the respondent’s drinking patterns against the safe drinking limits established by the Swedish National Institute of Public Health. Immediately after this followed comprehensive normative feedback with information describing the participant’s alcohol use compared with that of Swedish university students and, if applicable, personalized advice concerning the need for reducing any unhealthy levels or pattern of consumption. The student viewed the feedback on screen and could print it out. In addition the student received an email with a pdf file of the feedback.

### Measurements

In the baseline assessment, used in the per-protocol analysis, we calculated the average weekly consumption, in a typical week, as the number of standard glasses day by day. Frequency of heavy episodic drinking was assessed as at most once a month, 2–3 times a month, 1–2 times a week, or 3 or more times a week. Risky drinking at baseline was defined as either having risky weekly consumption (more than 14 standard glasses a week for men or more than 9 glasses a week for women, with 1 standard glass constituting 12 g of pure alcohol) or engaged in heavy episodic drinking (5 or more standard glasses for men, or 4 or more for women) 2 times a month or more often.

In the intention-to-treat analysis we assessed risky drinking at follow-up with the AUDIT questionnaire [30], asking the students about their drinking habits during the last 4 weeks. Students with an AUDIT score ≥8 for men and ≥6 for women were considered risky drinkers. AUDIT problem score was calculated as the sum of questions 7-10 of the AUDIT questionnaire, and AUDIT dependence score was calculated as the sum of questions 4-6. We used the third question in the AUDIT questionnaire concerning heavy episodic drinking separately in the analysis.

### Statistical Methods

We examined differences in proportions between groups with chi-squared tests. Weekly consumption of alcohol, total AUDIT score, AUDIT problem and dependence scores were logarithmically transformed (adding a constant of unit to handle zero values) and compared between groups by Student *t *test. The absolute change in weekly alcohol consumption (in the log-transformed scale) between baseline and follow-up was compared between groups with multilevel linear regression (where occasions were nested within individuals). The relative change in weekly alcohol consumption between baseline and follow-up was compared between groups by Mann-Whitney *U *test. All tests were performed 2-sided at *P *< .05. The statistical analyses were performed using SPSS version 19 (IBM Corporation, Somers, NY, USA) and Stata version 12 (StataCorp LP, College Station, TX, USA).

## Results

### Overall Response Rate

The response rate to the baseline survey was similar (approximately 37%) in groups 1 and 2. At follow-up, however, attrition was higher in groups 1 and 2 than in group 3 (Table 1). Thus, the response rate for the follow-up survey was approximately 52% in group 3 compared with around 41% in group 1 and 2 (*P *< .001, χ^2^_2 _= 54.6). The proportion who were risky drinkers at baseline was similar in groups 1 and 2 (around 56%) as well as in all groups at follow-up (around 50%) (Table 1).

**Table 1:** Attrition rates of all groups: group 1 (assessment and feedback), group 2 (assessment-only), and group 3 (no contact).

Timeline		Group 1	Group 2	Group 3	Total
**Baseline**					
	No. randomly assigned	1742	1742	1743	5227
	Completed baseline test, n (%)	631 (36.2%)	649 (37.3%)	NA^a^	1280 (36.7%)
	Risky drinkers, n (%)^b^	331 (57.2%)	326 (55.6%)	NA	657 (56.4%)
**Follow-up (for intention-to-treat analyses)**					
	Completed follow-up, n (%)	697 (40.0%)	737 (42.3%)	902 (51.7%)	2336 (44.7%)
	Risky drinkers, n (%)^c^	354 (50.8%)	364 (49.4%)	454 (50.3%)	1172 (50.2%)
**Follow-up (for per-protocol analyses)**					
	Risky drinkers, n (%)^c^	201 (57.4%)	207 (54.9%)	NA	408 (56.1%)

^a ^Not applicable.

^b ^Risky drinking was defined as having either a risky weekly consumption (>14 standard drinks/week for men or >9/week for women, with 1 standard drink constituting 12 g of pure alcohol) or engaged in a heavy episodic drinking (≥5 standard drinks for men or ≥4 for women) ≥2 times a month.

^c ^Risky drinking was defined as having an Alcohol Use Disorders Identification Test (AUDIT) score of ≥8 for men and ≥6 for women.

### Intention-to-Treat Analyses of All Groups

The sociodemographic characteristics of the participants in the follow-up from all three groups did not differ with regard to sex, age, and university section (see Table 2). The intention-to-treat analyses included all participants from all three groups who answered the follow-up survey. No alcohol parameters differed significantly between the three groups (see Table 3).

**Table 2:** Intention-to-treat analyses^a ^of groups 1, 2, and 3^b ^(total n = 2336): sample characteristics.

Characteristic		Group 1 (n = 697)	Group 2 (n = 737)	Group 3 (n = 902)	χ^2 ^(*df*)	*P *value
**Gender, n (%)**						
	Male	324 (46.5%)	336 (45.6%)	446 (49.4%)	2.71 (2)	.26
	Female	373 (53.5%)	401 (54.4%)	456 (50.6%)		
**Age (years), n (%)**						
	18–20	308 (44.2%)	352 (47.8%)	419 (46.5%)	3.75 (4)	.44
	21–25	292 (41.9%)	303 (41.1%)	363 (40.2%)		
	≥26	97 (13.9%)	82 (11.1%)	120 (13.3%)		
**University faculty, n (%)**						
	Arts and sciences	209 (30.0%)	197 (26.7%)	250 (27.7%)	4.66 (6)	.59
	Technology	299 (42.9%)	344 (46.7%)	411 (45.6%)		
	Education	91 (13.1%)	82 (11.1%)	115 (12.7%)		
	Health sciences	98 (14.1%)	114 (15.5%)	126 (14.0%)		

^a ^Including all who responded at follow-up and irrespective of drinking status and baseline participation.

^b ^Group 1 (assessment and feedback), group 2 (assessment-only), and group 3 (no-contact control).

**Table 3:** Intention-to-treat analyses of groups 1, 2, and 3 (total n = 2336): sample characteristics at follow-up and pairwise comparisons between groups 1 and 3.

Alcohol parameter		Group 1 (n = 697)	Group 2 (n = 737)	Group 3 (n = 902)	1 vs 2	1 vs 3	2 vs 3	
					Statistic (*df*) *P* value	Statistic (*df*) *P* value	Statistic (*df*) *P* value	
**AUDIT** ^a ^ **score**								
	Total, mean (SD)	7.3 (5.9)	6.9 (5.5)	7.3 (5.9)	1.31^b ^(1432) .19	0.29^b ^(1597) .77	–1.09^b ^(1637) .28
	≥8 (men)/6 (women) (n, %)	354 (50.8%)	364 (49.4%)	454 (50.3%)	0.28^c ^(1) .6	0.03^c ^(1) .9	0.14^c ^(1) .7
	Problem score (mean, SD)	1.8 (2.7)	1.6 (2.4)	1.8 (2.6)	1.81^b ^(1432) .07	0.50^b ^(1597) .62	–1.41^c ^(1637) .2
	Dependence score (mean, SD)	0.8 (1.4)	0.7 (1.2)	0.8 (1.4)	1.21^b ^(1432) .23	–0.16^b ^(1597) .87	–1.44^c ^(1637) .2
Weekly consumption (g), mean (median)		79.8 (48.0)	79.7 (48.0)	86.0 (48.0)	0.55^b ^(1432) .58	–0.25^b ^(1597) .80	–0.83^b ^(1637) .41
**Frequency of monthly heavy episodic drinking**								
	Never	171 (24.5%)	189 (25.6%)	244 (27.1%)	2.61^c ^(4) .6	4.70^c ^(4) .3	0.78^c ^(4) .9
	Less than monthly	158 (22.7%)	171 (23.2%)	196 (21.7%)			
	Monthly	249 (35.7%)	238 (32.3%)	288 (31.9%)			
	Weekly	117 (16.8%)	138 (18.7%)	173 (19.2%)			
	Daily/almost daily	2 (0.3%)	1 (0.1%)	1 (0.1%)			

^a ^Alcohol Use Disorders Identification Test.

^b^ *t *test.

^c ^χ^2^.

### Per-Protocol Analyses of Groups 1 and 2

The per-protocol analyses of groups 1 and 2 assessed outcomes among those participants with risky drinking at baseline who also answered the follow-up survey. Thus, 201 (57.4%) participants of the 377 who provided follow-up data from group 1 also provided data from the baseline survey, and similarly 207 (54.9%) of 421 from group 2 were included in the per-protocol analysis. There were no significant differences between groups 1 and 2 in the sociodemographic characteristics of the participants in the baseline survey. Weekly alcohol consumption at baseline was similar in both group with a mean consumption of 135.9 g/week for group 1 and 133.4 g/week for group 2 (median 120 g/week in both groups). Frequency of heavy episodic drinking did not differ significantly between the two groups at baseline.

Table 4 displays the results of the per-protocol analyses, showing one statistically significant difference in the relative change in weekly consumption between baseline and follow-up (*P *= .03); the absolute change in this outcome was not statistically significant. There were no other statistically significant differences between groups 1 and 2. The majority of risky drinkers in both groups reported drinking in a nonrisky way at follow-up.

**Table 4:** Per-protocol analyses of group 1 (assessment and feedback) and group 2 (assessment-only) (n = 408).

		Group 1 (n = 201)	Group 2 (n = 207)	Statistic (*df*)	*P *value
**Weekly alcohol consumption**						
	Weekly consumption^a ^(g) at follow-up (g), mean (median)	131.4 (108.0)	143.3 (108.0)	–1.12 (406)^b^	.26
	Absolute change in average weekly consumption (g) between baseline and follow-up, mean (median)	–4.5 (–12.0)	9.9 (0.0)	1.48^c^	.14
	Relative change (%) in average weekly consumption between baseline and follow-up, mean (median)	8.3 (–14.3)	20.8 (0.0)	38487^d^	.03
**Distribution of heavy episodic drinking occasions at follow-up**						
	Never	2 (1.0%)	3 (1.4%)	5.0 (4)^e^	.29
	Less than monthly	21 (10.4%)	22 (10.6%)		
	Monthly	109 (54.2%)	97 (46.9%)		
	Weekly	67 (33.3%)	85 (41.1%)		
	Daily or almost daily	2 (1.0%)	0 (0.0%)		
**AUDIT** ^f ^ **score at follow-up**						
	Total score, mean (SD)	11.6 (5.8)	11.0 (4.9)	0.65 (406)^b^	.52
	Score ≥8 (men)/6 (women), n (%)	168 (83.6%)	177 (85.5%)	0.3 (1)^e^	.59
	Problem score, mean (SD)	3.3 (3.3)	2.8 (2.9)	1.38 (406)^b^	.17
	Dependence score, mean (SD)	1.5 (1.9)	1.2 (1.4)	0.75 (406)^b^	.45
	Changed from risk to no risk, n (%)	118 (58.7%)	108 (52.2%)	1.8 (1)^e^	.18

^a ^Calculated from Alcohol Use Disorders Identification Test (AUDIT) questionnaire questions 1 and 2.

^b^ *t *test.

^c^ *z *test.

^d^ *U *test.

^e ^χ^2^.

^f ^Alcohol Use Disorders Identification Test.

## Discussion

This study found no between-group differences, indicating an absence of evidence of assessment or feedback benefit in the intention-to-treat analyses among unselected university students. There was one between-group difference among those who were risky drinkers at study entry in the per-protocol analysis. This potentially provides some very modest evidence of benefit attributable to receiving feedback in addition to assessment, although this should be interpreted in the context of the wider study finding of no effects.

There are three principal obstacles to making more definitive statements attesting to evidence of intervention ineffectiveness in the present study. First, the differential attrition between group 3 and groups 1 and 2 suggests problems of nonequivalence between the groups and thus bias in direct comparisons. Second, the present study was undertaken to prepare for a subsequent large trial and was thus not originally designed to produce such conclusions. Lastly, the approach taken to outcomes evaluation in which populations are randomized and compared regardless of their need for intervention should be carefully considered. Each of these issues shall be addressed in some detail.

Because of the randomized nature of this study, it can be inferred that differential attrition was caused by the earlier involvement of groups 1 and 2 than of group3 with the study. The earlier invitation to participate in the alcohol e-SBI was apparently not sufficiently different from the later alcohol survey, or the mere fact of a second alcohol-related email invitation may have interfered with the likelihood of accepting the invitation. Whatever the precise mechanism, the main implication is clear: selection bias is possible, if not likely, and outcomes for group 3 cannot be validly assumed to be directly comparable with those for groups 1 and 2 in relation to intervention effectiveness. Because of the lack of contact with group 3, the basis for making comparisons to evaluate this possibility is limited. This is restricted to the unlikelihood of data being collected at follow-up having been altered differentially between groups by the passage of time. Here there is evidence of a slight tendency for a greater proportion of male participants, and the presence of other unmeasured confounders cannot be ruled out. It should be noted that this issue of differential attrition particularly complicates randomized comparisons involving group 3 and is less of a problem for comparisons restricted to groups 1 and 2 only. A stronger conclusion can thus be drawn in relation to whether feedback added to the effects of assessment-only, and there is little evidence here that it did, notwithstanding one statistically significant difference among the per-protocol analysis outcomes.

The second reason for caution relates to the highly naturalistic Internet study context and the need to confront the difficult methodological challenges that this implies during the course of a long-term research program. Attrition has been a major source of difficulty in previous work in developing e-SBI in Linköping and other Swedish universities [31].

It is also a significant problem in the conduct of online trials in other populations as underscored by Eysenbach [32] and others [33]. The initial take-up of the routine service provision of e-SBI has been similar to that observed here and is likely to be influenced by factors such as patterns of email use, rates of risky drinking, the salience of alcohol, and interest in intervention. In Sweden, as elsewhere, there are also seasonal influences on drinking, including proximity to exams, and these complicate any analyses of change over time. Randomization, it should be noted, safeguards the validity of between-group comparisons, if attrition and other similar sources of bias are equivalently distributed between groups [34]. In the previous follow-up studies undertaken at Linköping University, less than half of those who participated at baseline did so at first follow-up, and approximately one-quarter participated in second follow-ups [19,34]. Here, rather than follow-up emails being sent by the student health care service as was done previously, blinding of participants to trial conduct was implemented. This involved an explicit attempt to separate the experience of follow-up from earlier e-SBI delivery. An email was sent by the first author (PB) requesting participation in a survey of student alcohol consumption, partially following the approach of Kypri and colleagues, who invited participation in a series of surveys at the outset and who obtained high follow-up rates [20]. As has been seen, this innovation, along with the use of cinema ticket incentives, was partially successful in restricting attrition at follow-up. It also introduced differential attrition as has been discussed. To rectify this, we have decided that in the next trial we will abbreviate the alcohol outcome measures and conceal them within a lifestyle questionnaire in the follow-up study. The overall attrition rate could be further improved with the use of stronger incentives, though this would potentially compromise the pragmatic nature of the study [35].

The third main reason for caution in drawing conclusions from the present study relates to our intention-to-treat approach to outcomes evaluation, which was highly conservative. The intervention comprised an automated email providing a means of accessing a website in an unselected population with an elevated prevalence of hazardous and harmful drinking. Thus, the intervention was delivered more widely than was necessary, as we only wished to intervene with risky drinkers. The intervention could be defined more narrowly as being delivered to those who accessed the website, with the email merely being the means of recruitment. Even if this definition is applied, the intervention would still have been accessed by students whose drinking was not risky and who would thus not have been deemed to merit individual targeting for intervention. More narrowly still, outcome evaluation could have been restricted to those whose drinking was found to be risky. The overarching problem is that a greater number of people were randomly assigned than would have been targeted for intervention. The primary rationale for proceeding in this way was that assessing eligibility, baseline data collection, and intervention delivery were all quickly integrated in 1 brief online session. We shaped our research design pragmatically around the real-world intervention opportunity, matching the research study to routine practice as it is delivered, rather than interfering with it for research purposes, which would have introduced external validity problems. This approach takes advantage of an opportunity to avoid any research participation effects that may be associated with screening and other aspects of study entry, in much the same way as cluster randomized trials can be used for this purpose. The obvious major disadvantage of this approach is that it biases hypothesis testing toward the null and thus is highly conservative. Thinking about outcomes evaluation needs to take account of these issues.

There then arises the question of the consistency of study findings with the existing literature. Put simply, there are no existing studies against which to compare our intention-to-treat findings, as none have used no-contact control groups. The per-protocol comparisons more closely reflect existing studies, and smaller between-group differences are observed. Thus, for both internal and external validity purposes, our test of the third hypothesis is particularly important. Comparisons with the existing literature also need to take account of the highly naturalistic study context. If our results are confirmed in further studies, they have important implications when considering the effectiveness of online alcohol interventions.

Our unusual study design confers many limitations, as well as strengths, some of which have already been considered. We used the AUDIT as an efficient summary measure of alcohol consumption and whether it may be hazardous or harmful. Although the AUDIT has been validated in online student contexts [9], this does not extend to use as an outcome measure in a trial. As well as uncertainty about such use, more direct behavioral measures of drinking may be better suited to universal prevention contexts. As the study was completely automated, there was no potential for subversion of randomization, nor of observer bias in ascertainment of study outcomes. The initial take-up or reach of the intervention is neither a simple strength nor limitation of this study, being part of the object of evaluation. Necessarily, the outcomes were self-reported and, although computerized data collection may minimize social desirability bias, the validity of self-reported outcome data in brief alcohol intervention trials needs to be studied. The approach used here involves deception, and therefore it is appropriate to consider whether less-ethically problematic methods could be used. For example, if we were only concerned with constraining assessment reactivity, would it not have been possible to adopt informed consent procedures and simply withhold assessment? This would indeed have been possible had we been interested only in exploring assessment reactivity effects. We are aware, however, of the potential for other research participation effects [29] and specifically wished to control for this possibility here. This need requires novel or underused approaches to research design, for example, and studies may involve avoiding informed consent [36]. As well as developing research methods in this program of study, we are very conscious of the need both to undertake ethical analyses in parallel and to undertake dedicated empirical studies to assist ethical evaluations. Further trials that provide access to large samples are likely to be useful for further substantive effectiveness trials, along with dedicated methodological and ethical studies of the issues contended with here.

## Multimedia Appendix 1

CONSORT ehealth checklist V1.6.1 [37].

## Abbreviations

AUDIT: Alcohol Use Disorders Identification Test
e-SBI: electronic screening and brief intervention
