# Supplementary material for: Effectiveness of a Proactive Mail-Based Alcohol Internet Intervention for University Students: Dismantling the Assessment and Feedback Components in a Randomized Controlled Trial
Source: J Med Internet Res. 2012 Oct 31;14(5):e142. doi: 10.2196/jmir.2062 (PMC3510746; doi:10.2196/jmir.2062)
Supplement: Supplementary file 1 [file jmir_v14i5e142_app1.pdf]

CONSORT-EHEALTH Checklist V1.6 Report	Manuscript Number	2062
Date completed	8/25/2011 14:50:29	
by	Preben Bendtsen	
RCT of the effectiveness of proactive mail based alcohol Internet intervention with university students: dismantling the assessment and feedback components		
TITLE		
**1a-i) Identify the mode of delivery in the title**		
"mail based alcohol Internet interventions "		
**1a-ii) Non-web-based components or important co-interventions in title**		
"All interventions were fully automated and did not involve any human involvement."		
**1a-iii) Primary condition or target group in the title**		
"University students"		
ABSTRACT		
**1b-i) Key features/functionalities/components of the intervention and comparator in the METHODS section of the ABSTRACT**		
"personalized normative motivating feedback on alcohol consumption with suggestion on how to decrease drinking"		
**1b-ii) Level of human involvement in the METHODS section of the ABSTRACT**		
"All interventions were fully automated and did not involve any human involvement.		
**1b-iii) Open vs. closed, web-based (self-assessment) vs. face-to-face assessments in the METHODS section of the ABSTRACT**		
"Mail based invitation to all freshmen"		
**1b-iv) RESULTS section in abstract must contain use data**		
"Overall, 45% (n=2,336) of those targeted for study completed follow-up. Attrition was similar in Groups 1 and 2 (approximately 41% retained) and lower in Group 3 (52% providing data). Intention-to-treat analyses among all participants regardless of their baseline drinking status revealed no differences between groups in all alcohol parameters at the 2 month follow-up. Per-protocol analyses of Groups 1 and 2 among those who accepted the e-mail intervention (approximately 37% of all offered) and who were risky drinkers at baseline (62% follow-up rate) suggested possible small beneficial effects on weekly consumption attributable to feedback"		
**1b-v) CONCLUSIONS/DISCUSSION in abstract for negative trials**		
"Electronic mail offer alone of alcohol intervention among unselected populations of university students was not found to be beneficial, though between-group differences in attrition prevent strong conclusions. This approach to outcome evaluation may be highly conservative and small benefits may follow actual uptake of feedback intervention in students who are risky drinkers, the actual target group"		
INTRODUCTION		
**2a-i) Problem and the type of system/solution**		
"The study addresses several of the identified gaps in previous research. The overall aim of this study was to evaluate the effectiveness of e-SBI, employing an RCT design that takes account of baseline assessment reactivity (and other possible effects of the research process) due to the similarity between the intervention and assessment content. The design of the study will allow exploration of the magnitude of the assessment effects per se. "		
**2a-ii) Scientific background, rationale: What is known about the (type of) system**		
"More research is needed on many aspects of e-SBI in student populations. The majority of existing research has been conducted in the USA. Many previous studies have required respondents to participate e-SBIs taking place in controlled settings, rather than allowing students to access e-SBIs using their own computers . Only a few published studies have described projects that made more comprehensive use of electronic media, by recruiting large numbers of participants via email, and having participants complete e-SBIs at their own convenience, using their own, or others' computers. A substantial number of e-SBI projects, including our own studies have been more feasibility studies and not been performed as full scale RCTs."		
METHODS		
**3a) CONSORT**		
"The overall aim of this study was to evaluate the effectiveness of e-SBI, employing an RCT design that takes account of baseline assessment reactivity (and other possible effects of the research process) due to the similarity between the intervention and assessment content. The design of the study will allow exploration of the magnitude of the assessment effects per se. "		
**3b-i) Bug fixes, Downtimes, Content Changes**		
"Consequently the system is stable and no bug fixes or content changes was necessary during the trial period and we observed no downtime of the system. "		

**4a-i) Computer / Internet literacy**		
No since this is after all a precondition to study at the university		
**4a-ii) Open vs. closed, web-based vs. face-to-face assessments:**		
"Students received an e-mail from the student health care service with a short welcome greeting to the university followed by an invitation to perform the Internet alcohol intervention by clicking on a hyperlink to the test. Two reminders were sent one week apart to those who had not answered and thereafter the link was closed. The link could only be used once in order to ensure that each student only performed the test once"		
**4a-iii) Information giving during recruitment**		
"Groups 1 and 2 were unaware that they were participating in a research study when they responded to the initial e-mails. Both groups expected that these e-mails were provided as routine practice by the student health care centre to help students think about their drinking. All three groups were unaware they were participating in an intervention study, and that they had been randomized. At follow-up, no explanation of the true nature of the study was given to students. Instead they were invited to participate in a seemingly unrelated student alcohol survey"		
**4b-i) Report if outcomes were (self-)assessed through online questionnaires**		
yes clearly stated		
**4b-ii) Report how institutional affiliations are displayed**		
Yes, the baseline survey was send from the Student Health Care center and the follow-up was send from a researcher (Professor)		
**5-i) Mention names, credential, affiliations of the developers, sponsors, and owners**		
"Conflict of interest<br>PB and MB own a company that has developed the e-SBI used in this study."		
**5-ii) Describe the history/development process**		
"The study is based on an e-mail based Internet alcohol intervention (e-SBI) that has been developed by the Lifestyle Intervention Research group (LIR group) at Linköping University ".		
**5-iii) Revisions and updating**		
"The computerized intervention is a fully automated single session intervention and have been used for some years as part of the daily routine in the great majority of Swedish Universities. Consequently the system is stable and no bug fixes or content changes was necessary during the trial period and we observed no downtime of the system."		
**5-iv) Quality assurance methods**		
**5-v) Ensure replicability by publishing the source code, and/or providing screenshots/screen-capture video, and/or providing flowcharts of the algorithms used**		
Reference to a demo web page of the intervention is given		
**5-vi) Digital preservation**		
No		
**5-vii) Access**		
Yes this is clearly stated in the paper		
**5-viii) Mode of delivery, features/functionalities/components of the intervention and comparator, and the theoretical framework**		
Clearly stated in varous parts of the methods section		
**5-ix) Describe use parameters**		
Single session intervention		
**5-x) Clarify the level of human involvement**		
No human contact		
**5-xi) Report any prompts/reminders used**		
No used in our study		
**5-xii) Describe any co-interventions (incl. training/support)**		
The interventions are already used routinely by staff in Student health care all over Sweden. The have not been trained to deliver the intervention besides having access to a manual and a web site from which they can administer the intervention.		
**6a-i) Online questionnaires: describe if they were validated for online use and apply CHERRIES items to describe how the questionnaires were designed/deployed**		
"The psychometric properties of existing screening instruments when administered online have been found to be reliable [7]. "		
**6a-ii) Describe whether and how "use" (including intensity of use/dosage) was defined/measured/monitored**		
Not applicable since tis was a single session intervention		

**6a-iii) Describe whether, how, and when qualitative feedback from participants was obtained**		
The participants could mail a comment or question to the researchers		
**7a-i) Describe whether and how expected attrition was taken into account when calculating the sample size**		
The study was a pilot study including all freshmen at one university. Ni power calculation was performed		
**7b) CONSORT**		
Not applicable		
**8a) CONSORT**		
"E-mail addresses were collected from the official university register in three separate data files. All participants has a 1/3 probability of allocation to any particular study condition. Randomisation was computerized and did not employ any strata or blocks. Each participant was given a random number between 0.0 and 1.0 with 5 decimals. For instance participant A might be given number 0.12345 and participant B might get 0.54321. The list of participants is then sorted in descending order by this number, and the list is cut into three equal parts (or as equal as possible depending on number of participants)"		
**8b) CONSORT**		
"Randomisation was computerized and did not employ any strata or blocks. "		
**9) CONSORT**		
"Each participant was given a random number between 0.0 and 1.0 with 5 decimals. For instance participant A might be given number 0.12345 and participant B might get 0.54321. The list of participants is then sorted in descending order by this number, and the list is cut into three equal parts (or as equal as possible depending on number of participants"		
**10) CONSORT**		
Done by one of the Authors responsible for sending the e-mails		
**11a-i) Specify who was blinded, and who wasn't**		
"Groups 1 and 2 were unaware that they were participating in a research study when they responded to the initial e-mails. Both groups expected that these e-mails were provided as routine practice by the student health care centre to help students think about their drinking. All three groups were unaware they were participating in an intervention study, and that they had been randomized. At follow-up, no explanation of the true nature of the study was given to students. Instead they were invited to participate in a seemingly unrelated student alcohol survey. As all study procedures were automated, the research team has no direct contact with study participants. "		
**11a-ii) Discuss e.g., whether participants knew which intervention was the "intervention of interest" and which one was the "comparator"**		
"Groups 1 and 2 were unaware that they were participating in a research study when they responded to the initial e-mails. Both groups expected that these e-mails were provided as routine practice by the student health care centre to help students think about their drinking. All three groups were unaware they were participating in an intervention study, and that they had been randomized. At follow-up, no explanation of the true nature of the study was given to students. Instead they were invited to participate in a seemingly unrelated student alcohol survey. As all study procedures were automated, the research team has no direct contact with study participants. "		
**11b) CONSORT**		
The difference is clearly stated		
**12a) CONSORT**		
No imputation was used.		
**12a-i) Imputation techniques to deal with attrition / missing values**		
No imputation was used.		
**12b) CONSORT**		
Not applicable		
RESULTS		
**13a) CONSORT**		
Yes clearly stated in table 1		
**13b) CONSORT**		
yes included in the paper		
**13b-i) Attrition diagram**		
yes included in the paper		
**14a) CONSORT**		
Yes clearly stated		
**14a-i) Indicate if critical "secular events" fell into the study period**		

yes clearly stated		
**14b) CONSORT**		
Performed as planned		
**15) CONSORT**		
Yes		
**15-i) Report demographics associated with digital divide issues**		
Yes		
**16-i) Report multiple “denominators” and provide definitions**		
yes		
**16-ii) Primary analysis should be intent-to-treat**		
Yes ITT analysis was performed as well as per protocol		
**17a) CONSORT**		
No		
**17a-i) Presentation of process outcomes such as metrics of use and intensity of use**		
Not applicable		
**17b) CONSORT**		
Not applicable		
**18) CONSORT**		
Yes		
**18-i) Subgroup analysis of comparing only users**		
Clearly stated		
**19) CONSORT**		
Yes		
**19-i) Include privacy breaches, technical problems**		
Clearly mentioned in the paper		
**19-ii) Include qualitative feedback from participants or observations from staff/researchers**		
During the trial period less than ten participants commented on the content of the intervention, giving mostly positive comments.		
DISCUSSION		
**20-i) Typical limitations in ehealth trials**		
More than half a page is discussing the limitations		
**21-i) Generalizability to other populations**		
"There then arises the question of the consistency of study findings with the existing literature. Put simply there are no existing studies against which to compare our ITT findings as there are not any no contact control groups in this area. The per protocol comparisons reflect more closely existing studies and smaller between-group differences are observed. These may be due to the highly naturalistic study context, and if confirmed in further studies, have important implications for consideration of the effectiveness of online alcohol interventions. "		
**21-ii) Discuss if there were elements in the RCT that would be different in a routine application setting**		
Nothing would be different. The intervention is already used routinely by student health care staff		
**22-i) Restate study questions and summarize the answers suggested by the data, starting with primary outcomes and process outcomes (use)**		
"The study revealed no between-group differences indicating an absence of evidence of assessment or feedback benefit in the intention-to-treat analyses among unselected university students. There were few between-group differences among those who were risky drinkers at study entry, potentially providing some very modest evidence of benefit attributable to the receipt of feedback in addition to assessment. "		
**22-ii) Highlight unanswered new questions, suggest future research**		
"Our unusual study design confers many limitations, as well as strengths, some of which have already been considered. Being completely automated there is no potential for subversion of randomisation, nor observer bias in ascertainment of study outcomes. Necessarily outcomes are self-reported and although computerised data collection may minimise social desirability bias, there is a need to study the validity of self-reported outcome data in brief alcohol intervention trials. Further trials which provide access to large samples are likely to be useful for this purpose, along with other methodological studies of the issues contended with here, as well as further substantive effectiveness trials. "		
Other information		
**23) CONSORT**		

Trial registration: ISRCTN24735383		
**24) CONSORT**		
Refer to a web page with a full version of the intervention.		
**25) CONSORT**		
"The study was funded by The Swedish Council For Working Life and Social Research (FAS, in Swedish). Grant number 2010-0024. JM is supported by a Wellcome Trust Research Career Development fellowship in Basic Biomedical Science (WT086516MA)."		
**X26-i) Comment on ethics committee approval**		
"The use of blinding and deception in this trial raises ethical issues. All students were subsequently offered the opportunity of feedback on their alcohol consumption at the time of the follow-up. Ethical approval for the study was given by the regional ethical committee in Linkoping, Sweden (Reference number: 2010/291-31). "		
**x26-ii) Outline informed consent procedures**		
Deception was used and thus the participants did not know they were in a trial		
**X26-iii) Safety and security procedures**		
No potentially harm is thought to be likely in this trial		
**X27-i) State the relation of the study team towards the system being evaluated**		
"Conflict of interest<br>PB and MB own a company that has developed the e-SBI used in this study."		
